# Assessment of Helminth Biodiversity in Wild Rats Using 18S rDNA Based Metagenomics

**DOI:** 10.1371/journal.pone.0110769

**Published:** 2014-10-23

**Authors:** Ryusei Tanaka, Akina Hino, Isheng J. Tsai, Juan Emilio Palomares-Rius, Ayako Yoshida, Yoshitoshi Ogura, Tetsuya Hayashi, Haruhiko Maruyama, Taisei Kikuchi

**Affiliations:** 1 Division of Parasitology, Faculty of Medicine, University of Miyazaki, Miyazaki, Japan; 2 Biodiversity Research Center, Academia Sinica, Taipei, Taiwan; 3 Division of Microbial Genomics, Department of Genomics and Bioenviromental Science, Frontier Science Research Center, University of Miyazaki, Miyazaki, Japan; Queensland Institute of Medical Research, Australia

## Abstract

Parasite diversity has important implications in several research fields including ecology, evolutionary biology and epidemiology. Wide-ranging analysis has been restricted because of the difficult, highly specialised and time-consuming processes involved in parasite identification. In this study, we assessed parasite diversity in wild rats using 18S rDNA-based metagenomics. 18S rDNA PCR products were sequenced using an Illumina MiSeq sequencer and the analysis of the sequences using the QIIME software successfully classified them into several parasite groups. The comparison of the results with those obtained using standard methods including microscopic observation of helminth parasites in the rat intestines and PCR amplification/sequencing of 18S rDNA from isolated single worms suggests that this new technique is reliable and useful to investigate parasite diversity.

## Introduction

Parasitism is one of the most common and successful lifestyles on the Earth [1] and has evolved independently at least 60 times during the evolutionary history of animal life [1] [2]. Several parasite lineages have diversified greatly over geological time. As a result, parasites outnumber their free-living relatives in some taxonomic groups in animal kingdom [1].

Studying parasite diversity is important for at least 3 major reasons. First, parasites are now recognised as playing important roles in ecosystem fractions [3] by influencing the populations and communities of their hosts [4]. Second, many parasite species are medically and agriculturally important. Although little is known about their evolutionary origins, several human parasites may have evolved by switching to humans from wild or domestic animals [5], [6]. Additionally, species interactions involving parasites are a key to understanding many biological invasions and emerging infectious diseases [3]. Finally, because of the many independent transitions to parasitism within taxonomic groups, researchers can study the processes of evolution as the phenomena is related to speciation rates and diversification [1]. Therefore, the number of studies investigating the patterns of parasite diversity among/within host species and among geographical regions has been increasing in recent years [4]. However, the traditional approach of identifying all individual parasitic worms based on microscopic observation and PCR amplification/sequencing of 18S rDNA from isolated single parasites is time consuming, and requires highly specialised experience in morphology. In addition, morphological identification is simply impossible in some cases. As a result, parasite communities are not well classified, leaving diversity analysis ambiguous and non-holistic. Recent advances in high-throughput massively parallel sequencing, also called ‘next generation sequencing’ (NGS), are revolutionising the description of microbial diversity within and across complex biomes from the human body to the Earth’s biosphere [7], [8]. The greater sequence coverage and lower per-base sequence cost offered by NGS instruments including Illumina sequencers and 454 pyrosequencers have been greatly contributing to this progress.

Most of the metagenomic studies performed to date have targeted the biodiversity of prokaryotic communities using 16S ribosomal RNA gene (rDNA) sequences [9], [10], [11]. Attempts to assess eukaryotic diversity using NGS techniques have just begun for fungi [12], nematodes [13], [14] and marine microbes [15]. In this study, we performed eukaryotic 18S rDNA-based metagenomics to assess biodiversity of helminth parasites (i.e. Nematoda, Cestoda and Trematoda) in the alimentary tract of wild rats. We analysed massive numbers of sequence reads obtained by 18S rDNA-PCR amplification followed by Illumina sequencing. To evaluate accuracy, sensitivity and resolution power of the method, we compared these results with those from the standard methods including extraction of helminth parasites from the intestine, microscopic observation and single-worm PCR amplification/sequencing. Our results suggest this new technique is useful for the identification of animal parasites and the assessment of parasite diversity.

## Materials and Methods

### Collecting wild rats

Nine wild rats (7 *Rattus norvegicus* and 2 *R. rattus*), which were captured under the rodent extermination programmes at 2 locations of Miyazaki City, Japan, from November 2013 to January 2014 (Table 1) were received from Miyazaki City Phoenix Zoo or Miyazaki Pest Control Association. The rats were transported to the laboratory and maintained in clean cages for 12 hr before sacrificing by ether inhalation. Faecal pellets they excreted *ad libitum* were collected for metagenomic analysis.

**Table 1:** Wild rats used in this study.

Rat ID*	Species	Gender**	Body length (cm)	Tail length (cm)	Body weight (g)	Location	Collection Date
TR2	*Rattus norvegicus*	F	18	NA	NA	NishiTachibana St., MiyazakiCity	6-Nov-13
TR3	*Rattus norvegicus*	F	19	NA	NA	NishiTachibana St., MiyazakiCity	6-Nov-13
TR4	*Rattus norvegicus*	F	19	16	151.6	NishiTachibana St., MiyazakiCity	8-Nov-13
TR5	*Rattus norvegicus*	M	23	21	311.2	NishiTachibana St., MiyazakiCity	8-Nov-13
TR6	*Rattus norvegicus*	M	16.5	14.5	97.5	NishiTachibana St., MiyazakiCity	8-Nov-13
TR7	*Rattus norvegicus*	F	20.5	18	191.2	NishiTachibana St., MiyazakiCity	8-Nov-13
TR8	*Rattus norvegicus*	F	18	17	159	NishiTachibana St., MiyazakiCity	9-Nov-13
ZR1	*Rattus rattus*	F	14	18	100.8	Phoenix zoo, Miyazaki City	28-Jan-14
ZR2	*Rattus rattus*	M	14	15	96.3	Phoenix zoo, Miyazaki City	28-Jan-14

*Rat IDs were assigned based on collection locations (TR or ZR).

**F: female, M: male.

### Ethical statement

Animal experiments were performed in accordance with the procedures approved by the Animal Experiment Committee of the University of Miyazaki under an approval no. 2009-506-6, as specified in the Fundamental Guidelines for Proper Conduct of Animal Experiment and Related Activities in Academic Research Institutions under the jurisdiction of the Ministry of Education, Culture, Sports, Science and Technology, Japan, 2006.

### Isolation of parasitic worms from rat intestines

Parasites were isolated from rat intestines as previously described with some modification [16]. Briefly, whole intestines were extirpated from freshly sacrificed rats and separated into 2 parts (20 cm from the pylorus ring and the remainder). They were dispreaded, washed and incubated separately in PBS (phosphate buffered saline) at 37°C for 2 h to let worms emerge. PBS was then centrifuged at 3000×*g* for 5 min to concentrate the isolated parasites, which were observed using light microscopy.

### DNA extraction, sequencing from individual parasites and phylogenetic analyses

DNA from nematodes was extracted into DirectPCR Lysis Reagent (Viagen) containing 20 mM dithiothreitol (Wako) and 0.5 mg/mL proteinase K (Qiagen). Individual nematodes were transferred to a 10-µL aliquot of the lysis buffer and incubated at 60°C for 20 min followed by 95°C for 10 min. DNA extraction from cestodes was performed using the QIAamp DNA Mini Kit according to the manufacturer’s instructions (Qiagen). One microlitre of the extract was used for PCR amplification of the 18S ribosomal RNA gene. These PCR reactions contained primers 988F and 1912R [17] for nematodes and wormA and wormB for cestodes [18], along with GoTaq Green Master Mix (Promega). PCR products were purified using the MinElute 96 UF Kit (Qiagen) and sequenced using BigDye Terminator v3.1 and an ABI 3130 sequencer (Applied Biosystems).

Phylogenetic analyses were performed with obtained and published 18S rDNA sequences. Sequences were aligned with MAFFT v6.864b [19] using ‘–auto’ option, and the alignments were cleaned with Gblocks v.0.91b [20] using flags ‘−b4 = 10 −b5 = n −b6 = y −s = y’. Phylogenetic analyses were performed with RAxML v.7.2.8 [21]. The trees were bootstrapped 100 times for support.

### Illumina library construction and sequencing

DNA extraction from individual faecal pellets (approximately 0.1 g) obtained from wild rats as described above was performed using the PowerSoil DNA extraction kit (MoBio) as recommended in the Earth Microbiome Project (http://www.earthmicrobiome.org/). Barcoded PCR products were generated according to the protocol of the Earth Microbiome Project [22] (http://www.earthmicrobiome.org/emp-standard-protocols/18s/). Briefly, the V9 region of the eukaryotic 18S rRNA gene was amplified in triplicates for each sample with 1391f and EukBr primers [23] containing Illumina adaptors and a unique 12 bp Golay barcode using Ex Taq Polymerase (Takara). PCR amplification was performed in the presence of the mammal blocking oligo [24] on a 30-µL scale under the following conditions: 94°C for 3 min; 22 cycles of 94°C for 45 s; 65°C for 15 s; 57°C for 30 s; 72°C for 90 s and 72°C for 10 min. PCR products from the 3 reactions were combined and purified using the MinElute 96 UF PCR Purification Kit (Qiagen). The final sequencing library was prepared by mixing equal amounts of PCR products and purifying the mixture using agarose gel electrophoresis and the QIAquick Gel Extraction Kit (Qiagen).

Libraries were sequenced with Illumina MiSeq using the MiSeq Reagent Kit v2 (500cycles) (Illumina) and custom sequencing primers (http://www.earthmicrobiome.org/emp-standard-protocols/18s/) according to the manufacturer’s recommended protocol (https://icom.illumina.com/). The linearization, blocking and hybridization step was repeated in situ to regenerate clusters, release the second strand for sequencing and hybridise the R2 sequencing primer. This was followed by another 250 cycles of sequencing to produce paired-end reads. The sequencing data have been deposited to DDBJ sequence read archive (DRA) under the BioProject PRJDB3050.

### Illumina data analysis

Illumina sequence data was processed using QIIME version 1.8.0 [25]. Paired-end reads were joined using the ‘fastq-join’ method in QIIME (join_paired_ends.py). After QIIME quality filtering and library splitting according to the Golay barcode sequences (split_libraries_fastq.py: –store_qual_scores -q 9–max_barcode_errors 2–sequence_max_n 1–max_bad_run_length 2 −p 0.75), 18S rRNA OTUs were picked from the reads using a closed-reference OTU picking protocol against the SILVA 108 database (Eukarya_only) [26] at 95% identity with ‘uclust’ (pick_otus.py: –max_accepts 1–max_rejects 8–stepwords 8–word_length 8).

## Results

### Identification of parasites from rat intestines

**Figure 1:**
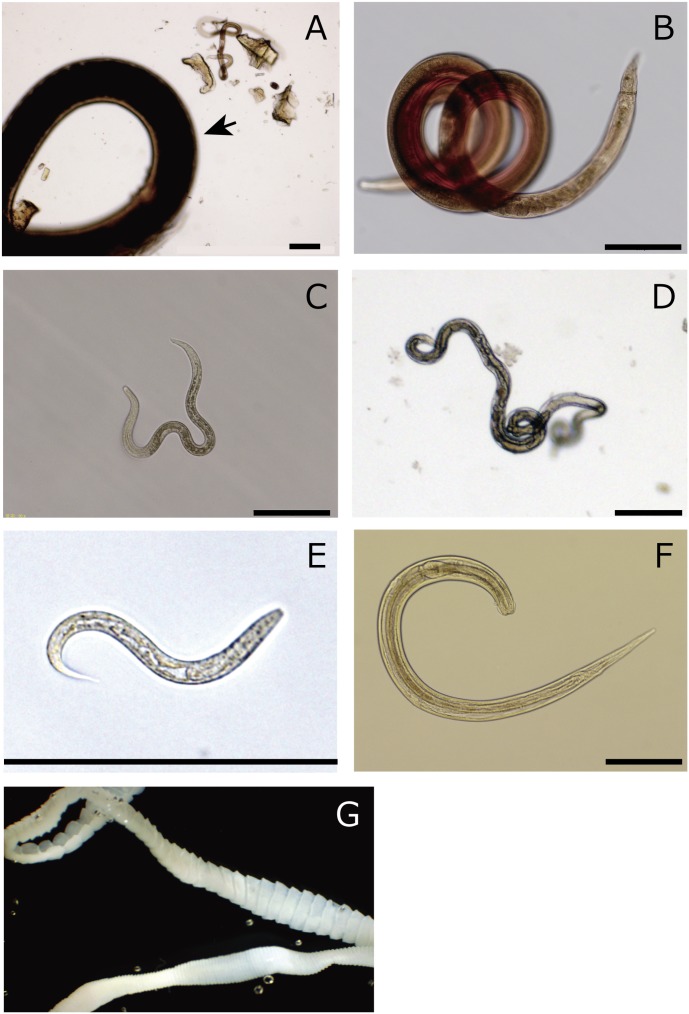
Seven morphological types identified in wild rat intestines. (A–F) Nematoda, (G) Cestoda (bar = 200 µm).

Seven and two wild rats were collected at 2 contrasting locations in Miyazaki City, Japan: a restaurant downtown in the middle of the city (TR) and a zoo in the suburbs (ZR), respectively (Table 1). The rat IDs were named with the header TR or ZR according to the location from where they were collected. The sample was composed of 3 males and 6 females from 2 species (*R. norvegicus* and *R. rattus*) with varying body sizes (body weight ranging from 95 g to over 300 g) (Table 1). The parasite isolation protocol from the intestines detected nematodes in 8 out of the 9 rats (Table 2). On the other hand, cestodes were identified in only 2 *R. rattus* rats collected in the zoo (*R. rattus*). They were more than 20 cm in length and difficult to distinguish from each other by their morphology (Table 2, Figure 1). The sequences of their 18S rRNA genes were highly similar (99.9% identity) to those of *Hymenolepis diminuta* (JX310720) (Table 3, 4, Figure S1E). We found no flukes in the wild rat intestines.

**Table 2:** Numbers of helminth parasites identified in rat intestines.

Rat ID	Nematodes						Cestodes
	morphA	morphB	morphC	morphD	morphE	morphF	
TR2	0	75 (35)	1 (1)	85 (40)	11 (5)	0	0
TR3	2 (0)	19 (19)	6 (3)	142 (84)	7 (2)	0	0
TR4	0	134 (117)	2 (0)	42 (33)	0	0	0
TR5	0	177 (177)	0	128 (125)	0	0	0
TR6	0	37 (37)	2 (1)	165 (160)	0	0	0
TR7	0	344 (333)	6 (4)	273 (234)	0	0	0
TR8	0	130 (117)	0	94 (74)	0	0	0
ZR1	0	0	0	0	0	0	3
ZR2	0	0	0	0	0	2 (0)	3

Helminth parasites observed in the rat intestines were classified into 7 groups based on their morphological traits (Figure 1).

The total number of parasites is shown in each cell. Values in parentheses are the number of parasites identified in the first 20 cm from the pylorus ring.

**Table 3:** Morphological characters of isolated helminths.

MorphologyType	No. measured	Stage	Body length(mm)^a^	Body width(µm)^a^	Length ofesophagus(µm)^a^	Descriptive characters
morph A	1	ND	210	294	ND	large body size, cylindrical shape,creamy-white colour
morph B	4	adult male	2.26 (1.74–2.76)	74.6(61.1–92.7)	247 (192–294)	red spiralled body, a prominentunbrella-like bursa and twospicules at the posterior end
	4	adult female	2.81(2.37–3.26)	68.71 (63.9–72.2)	269 (241–330)	red spiralled body, ellipsoidaleggs inside of the body, vulvaopens at the posterior end
morph C	4	larva	0.83 (0.77–0.88)	29.0(19.9–40.5)	178 (140–226)	middle size (∼1.0 mm)rhabditiform
morph D	3	adult female	1.98(1.83–2.10)	30.0(28.7–31.0)	147 (146–147)	thin body, long pharynx, onlyfemales found, ellipsoidal eggsand vulva in the middle of thebody
morph E	3	larva	0.19 (0.17–0.20)	14.4 (14.2–14.7)	66.2 (53.6–72.6)	small size (∼0.20 mm)rhabditiform
morph F	1	adult male	1.57	74.5	258	braod cervical alae, ovalesophageal bulb, slightly hookedtail with no clear spiclues
	1	unmatured female^b^	1.32	60.1	254	braod cervical alae, ovalesophageal bulb, pharynx plainlyvisible, conical shaped tail
morph G	2	adult	>200	1181 (1153–1231)	NA	flat segemented body, 4 suckers atthe scolex, proglottids with bothmale/female sexual organs

a mean; range of the size in parentheses.

b probably 4th stage lavae.

ND; Not determined, NA; Not applicable.

**Table 4:** Species identification of isolated helminths based on 18S rDNA sequencing.

Morphology type	Number of sequences	Top hit in nematode 18S database	Sequencesimilarity (%)	Alignmentlength (bp)	sequence ID in Fig. S1
morph A	1	*Ascaridia galli* [EF180058]	98.2	895	A1
morph B	26	*Nippostrongylus brasiliensis*[AJ920356]	99.2	906	B1
	12	[AJ920356] or *Heligmosomoides polygyrus* [AJ920355]	97.5	906	B2
morph C	6	*Nippostrongylus brasiliensis*[AJ920356]	99.2	906	C1
	3	[AJ920356] or *Heligmosomoides polygyrus*[AJ920355]	97.5	906	C2
	1	*Ascaridia galli* [EF180058]	98.1	906	C3
morph D	12	*Strongyloides venezuelensis*[AB923887]	99.7	902	D1
	14	*Strongyloides ratti* [AB923889]	99.8	895	D2
morph E	8	*Strongyloides ratti* [AB923889]	99.8	895	E1
morph F	2	*Aspiculuris tetraptera* [EF464551]	100	135	F1
morph G	2	*Hymenolepis diminuta* [JX310720]	99.9	2005	G1

### Nematode diversity

The total numbers of nematodes observed in each of the 8 rats varied, ranging from 2 in ZR2 to 623 in TR7. Most of the nematodes isolated were obtained from the first 20 cm of the intestine from the pylorus ring (Table 2).

Based on morphology, the nematodes were classified into 6 groups (Figure 1A–1F, Table 2). Briefly, morph A had a very large body size (more than 1 cm long) with a cylindrical shape and creamy-white colour, morph B had a differentiable red spiralled body, morph C had an medium body size (∼1.0 mm in length) among the observed nematodes and a rhabditiform morphology, morph D was thin, 2–3 mm in length and characterised by the presence of a long pharynx, morph E was a small rhabditiform, probably first- or second-stage larvae and morph F was 1.4–2.0 mm long and differentiable by prominent and broad cervical alae (Figure 1A–1F). Those morphological characteristics are summarised in Table 3.

The 18S rDNA sequencing analysis of individual nematode suggested that these morphological groups consisted of at least 6 distinct species including those belonging to the genera *Ascaridia, Nippostrongylus, Heligmosomoides, Strongyloides* and *Aspiculuris* (Table 4, Figure S1). Morphological descriptions of the species are mostly consistent with the morphological characteristics observed. The 18S rDNA sequences of *N. brasiliensis* and *H. polygyrus* were very similar to each other. In morph B and morph C samples, we found 2 distinct sequence groups showing high similarity to the *N. brasiliensis* 18S rDNA sequence (99.2% and 97.5% identity, respectively). But one of them also showed comparable high similarity to *H. polygyrus* (97.5% identity) (Table 4). In the maximum-likelihood tree, those two sequences were classified together into a cluster which consisted of *N. brasiliensis* and *H. polygyrus* sequences, but they were sub-clustered separately from each other (Fig. S1B).

These results suggested the rats collected in the restaurant downtown were heavily infected by multiple parasitic nematode species including *Ascaridia, Nippostrongylus* (or *Heligmosomoides*) and *Strongyloides* nematodes. Of those, the majority of the infections were from *N. brasiliensis* and *Strongyloides* species. In contrast, rats collected in the zoo showed infrequent nematode infection; only a pin worm species was detected (Table 2, 3, 4).

### 18S rDNA Illumina sequencing

DNA was extracted from faecal samples collected from individual rats. The variable regions (V9) of the eukaryotic 18S ribosomal RNA genes present in each faecal community were amplified by PCR, and the resulting amplicons were sequenced on an Illumina MiSeq using 500 cycles.

Of the 6 million Illumina reads from the V9 regions of the eukaryotic 18S rRNA genes that passed the QIIME quality filters and were correctly assigned to each sample on the basis of the barcode sequences, 80.6% matched reference sequences in the SILVA 108 database at 95% sequence identity. They clustered into a total of 391 Operational Taxonomic Units (OTUs).

**Figure 2:**
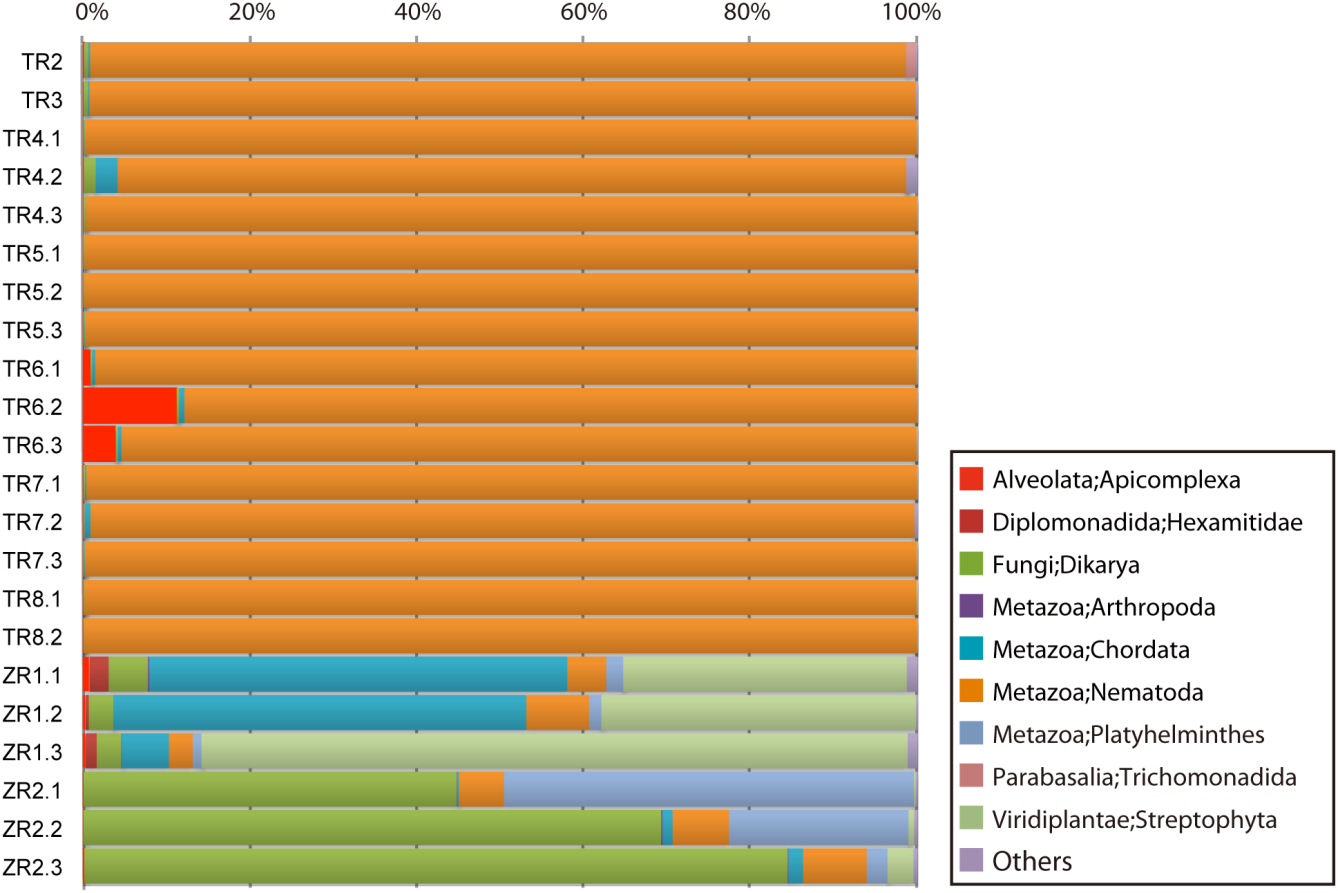
QIIME phylum-level classification of the 18S rDNA Illumina sequencing data.

The QIIME level-2 OTU classifications (phylum level) are shown in Figure 2. In TR samples, 90–99% of the reads were assigned to Nematoda sequences. In TR6 samples, approximately 10% of reads were assigned to Apicomplexa (Figure 2).

Less than 10% of the reads in ZR samples were assigned to nematodes. Reads that were assigned to Streptophyta (Planta), Chordata (Animalia) or Dikarya (Fungi) were more represented. Reads assigned to Platyhelminthes were also found in ZR samples (approximately 2% in ZR1 samples and 3–49% in ZR2 samples) (Figure 2). Deeper classification revealed that most of the Streptophyta reads in ZR samples were assigned to corn (*Zea*), Chordata reads were to assigned to pig (*Sus*) and Dikarya reads were assigned to yeast (*Saccharomyces* or *Candida*) (Table S1). Samples from each rat (different pieces of faecal pellets from the same day) showed similar contents although their ratios were different (Figure 2). Other taxa to which more than 0.1% of the reads were assigned in any sample included Hexamitidae (approximately 0.04%–2.3% in ZR1 samples), Arthropoda (>0.1% in ZR1 and ZR2, <0.1% in TR samples) and Trichomonadida (>0.1% TR2 and TR7 samples, <0.1% in other TR samples) (Figure 2).

**Figure 3:**
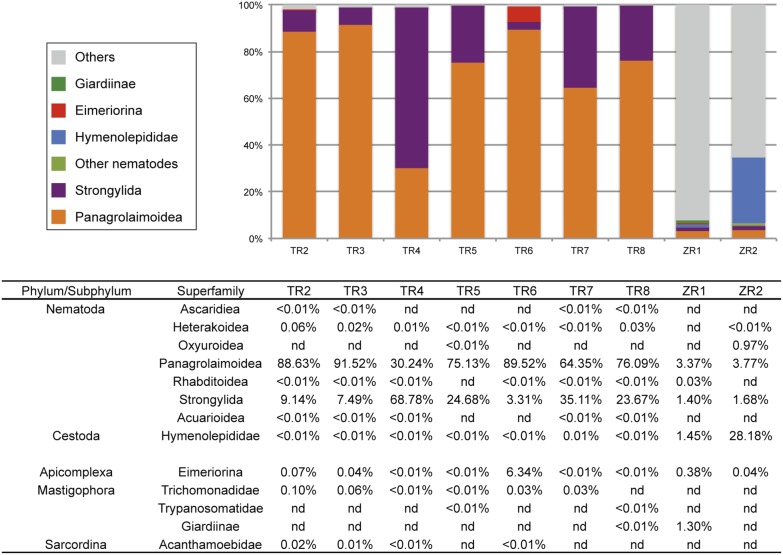
Parasite sequences in the QIIME superfamily-level classification.

The QIIME level-6 classifications (mostly superfamily level) clustered OTUs into 135 taxonomic groups (Table S1) and revealed that more than 90% of the total assigned reads in TR samples were classified into Panagrolaimoidea or Strongylida, which include *Strongyloides* species and *Nippostrongylus* species, respectively (Figure 3). Other nematode taxonomic groups in TR samples to which reads were assigned (with a minimum number of read filter of 4) include Ascaridiea, Heterakoidea, Oxyuroidea, Rhabditoidea and Acuarioidea although they were all rare OTUs (<0.1%, Figure 3, Table S1). In ZR samples, only 3 nematode superfamilies (Panagrolaimoidea, Strongylida and Oxyuroidea) had assigned reads (Figure 3, Table S1).

Because the SILVA 108 database contained only an essential number of nematodes reads, we sought to reclassify the reads assigned to Nematoda in the QIIME classification by BLAST similarity analysis using an in-house nematode 18S database. Although it was still difficult to classify them into deeper levels due to the short read length (∼150 bp), the database enabled us to get a better insight into the nematode community. The reads assigned to Panagrolaimoidea or Strongylida in the QIIME classification showed the highest similarity to *Strongyloides* species and *N. brasiliensis* sequences in the nematode 18S database, respectively (Table 5). The reads assigned to Ascaridiea, Heterakoidea or Acuarioidea were highly similar to each other and showed the greatest similarity to *Ascaridia galli* or *Heterakis gallinarum.* The reads assigned to Oxyuroidea were most similar to *Aspiculuris tetraptera* (Table 5). These results were mostly consistent with the results of the standard method (direct microscopic observation) (Table 2, 6). A trend was observed in the *Strongyloides*/*Nippostrongylus* ratio; samples with a lower ratio in the direct observation showed a lower reads ratio although *Strongyloides* reads ratios were always higher than the observed ratios (Table 2, 6, Figure 3). In addition to the species that we identified in the standard method, we found 18S rDNA reads which matched sequences from a free-living species *Oscheius spp.*

**Table 5:** Nematode reads re-assigned with in-house nematode 18S database.

Order; Family	TR2	TR3	TR4	TR5	TR6	TR7	TR8	ZR1	ZR2	Possible nematode species
Rhabditida; Strongyloididae	119998	500677	208730	1085734	862840	292994	477358	129	526	*Strongyloides spp.*
Strongylida; Heligmonellidae	12381	40979	474806	356714	31943	159843	148495	55	238	*N. brasiliensis, H. polygyrus*
Ascaridida; Ascaridiidae, Heterakidae	87	134	78	5	0	41	208	0	0	*A. galli, H. gallinarum*
Oxyurida; Heteroxynematidae	0	0	0	0	0	0	0	0	137	*A. tetraptera*
Rhabditida; Rhabditidae	0	0	27	0	0	0	0	0	0	*Oscheius sp*

Minimum 4-read filter was applied.

**Table 6:** Comparison of the detection results from the standard method and the 18S Illumina method.

		TR2		TR3		TR4		TR5		TR6		TR7		TR8		ZR1		ZR2	
Standard method species	Illumina best classification	Std	Ilm	Std	Ilm	Std	Ilm	Std	Ilm	Std	Ilm	Std	Ilm	Std	Ilm	Std	Ilm	Std	Ilm
*A. galli, H. gallinarum*	*A. galli, H. gallinarum*			+	+														
*A. tetraptera*	*A. tetraptera*																	+	+
*N. brasiliensis* a)	*N. brasiliensis, H. polygyrus*	+	+	+	+	+	+	+	+	+	+	+	+	+	+				
*S. ratti*	*Strongyloides* sp.	+	+	+	+	+	+	+	+	+	+	+	+	+	+				
*S. venezuelensis*	*Strongyloides* sp.	+	+	+	+	+	+	+	+	+	+	+	+	+	+				
	*Oscheius* sp.					-	+												
*H. diminuta*	*Hymenolepis* sp.															+	+	+	+

“+” indicates worm/sequence detected. Std; Standard method, Ilm; 18S Illumina method.

a) these samples can be divided into two groups based on the 18S sequences.

The Platyhelminthes reads both in TR and ZR samples were all classified into Hymenolepididae at the superfamily-level classification. The ZR2 rat had a higher percentage of Platyhelminthes reads (28%) than the ZR1 rat while they were rare in TR samples (<0.1%).

Reads assigned to the taxa that included parasitic Protozoa species were also identified in the QIIME classifications. Reads which were classified into Eimeriorina and Trichomonadidae were found in several samples (Figure 3). Notably, as much as 6.34% of the reads were classified into Eimeriorina in TR6 (Figure 3). Reads that were assigned to Giardiinae were found in TR8 and ZR1 samples (<0.01% and 1.30%, respectively), Trypanosomatidae in TR5 and TR8 and Acanthamoebidae in TR samples although they were rare (<0.1%) (Figure 3).

## Discussion

Although ‘metagenomics-based’ studies of bacterial communities using 16S rDNA sequences have been extensively performed recently, there have been only few metagenomics reports targeting eukaryotic communities. In this study, we showed the power and usefulness of 18S rDNA Illumina sequencing for population studies of eukaryote parasites. Compared with traditional methods (isolation from rat intestines, microscopic observations and single-worm sequencing of 18S rDNA), the 18S metagenomics-based method is easy, quick to apply and sensitive. The 18S metagenomics-based method requires only faecal samples (no need to sacrifice hosts) and requires neither special techniques nor knowledge of parasite morphologies. Most importantly, 18S Illumina sequencing identified more varieties of parasite than the traditional methods in addition to all of the parasites identified using the traditional method.

The amount of parasite DNA in a faecal sample is expected to vary widely depending on their life cycles or conditions. The higher prevalence of *Strongyloides* species in the Illumina sequencing results than that in the result of the standard technique may have arisen because they produced and excreted more eggs/larvae into faeces than those by the other parasite species. We found a large Ascaridia-like nematode in the intestine of one of the rats (Figure 1). However, the number of reads that were assigned to this nematode group (Ascaridiae) or a closely related group (Heterakoidea) in level 6 QIIME was very small (Figure 3). This suggests that the nematode was not very active in the rat body and did not produce eggs, possibly because it was sexually immature. Ascaridia/Heterakoidea nematodes are known as parasites of birds [27], and as far as we know, no Ascaridia/Heterakoidea nematodes have been reported from rodents. Therefore, the presence of Ascaridia/Heterakoidea nematode might reflect a recent accidental swallow of the parasite by the rat.

DNA extraction efficiencies from eggs or larvae of each species can also affect the number of sequence reads obtained. To reduce this kind of effects we used a well-established DNA extraction protocol using MoBio PowerSoil kit, which was used as a standard method in the Earth Microbiome Project (http://www.earthmicrobiome.org/) and the Human Microbiome project [28]. The protocol, combining mechanical and chemical sample disruptions, has been widely used for various types of samples including soils and faeces, and it was shown that has been shown capable to extract DNA even from tough organisms such as spores or fungal mats (http://www.mobio.com/). The bias due to extraction efficiency in this study, therefore, can be low, but tests and optimisations of DNA extraction methods from parasites are still needed.

It is an interesting challenge to quantify parasites using 18S Illumina reads. To achieve the estimate, at least 2 normalisation steps seem to be needed. First, absolute read numbers need to be normalised using control DNA. Because DNA extracted from faeces was amplified by PCR and then appropriate amounts of the products for each sample were mixed and used for sequencing, we should not simply use the read numbers to estimate the amount of each parasite’s DNA. Control DNA can be endogenous or exogenous (i.e. artificially added to each sample). For example, a large number of reads were assigned to corn (*Zea*) or pig (*Sus*) (Figure 3) in ZR samples. These probably resulted from the DNA of foods that the rats ingested. These reads were also present in TR samples although the ratios of read numbers were very small (<0.01%). These kinds of reads, which can be assumed to always exist in relatively fixed amounts, could be a candidate for endogenous control DNA. Second, we need to use ‘species factors’ that are calculated on the basis of DNA emission in faeces per individual parasite to estimate the number of parasites in a host body. In addition, copy number differences of rRNA genes between each parasite genome are also a subject to be considered [29].

We used the SILVA 108 database as a reference to classify 18S Illumina reads in QIIME. This study showed the database is very useful to provide a rough estimate of the structure of the helminth community in host bodies. However, the nematode and cestode sequences in the SILVA database were limited to only essential sequences and were not broad enough to cover nematode and cestode diversities. For example, the 18S rDNA sequences of rat parasites like *S. ratti*, *N. brasiliensis* and *H. polygyrus* do not exist in the SILVA 108 database. Therefore, we used an in-house nematode 18S rDNA database to obtain a better insight into the community structures. Additionally, because Illumina sequencing read lengths have been getting longer (up to 300 bp paired-end with the v3 kit), it will be able to use a region of the 18S rRNA gene longer than the V9 region (∼150 bp) used in this study to obtain a better resolution during the classification step. Attempts to develop better primers to amplify and classify eukaryote organisms by metagenomics have already started [30], [31]. Because parasites have considerably diverged within some small taxonomic groups and parasite species that researchers want to distinguish are often closely related to each other, superfamily-level classifications achievable with the QIIME software are sometimes not sufficient enough in depth to make fine distinctions. Therefore, developing a more complete database and using longer 18S sequencing reads are the next key steps to improve this technique as a more powerful tool to study parasite community structures.

Another important finding is that we identified a number of Eimeriorina sequences in the 18S Illumina data from TR rats (6.3% of total reads in TR6). Eimeriorina contains *Eimeria* species, which are protozoan parasites of animals [32]. These sequences were likely from parasites that have infected the rats. We also identified sequences that were assigned to taxa which include parasitic Protozoa species including *Trichomonas*, *Giardiia*, *Trypanosoma* and *Acanthamoeba* spp. This suggests 18S Illunima sequencing is also useful to study protozoan parasite community structures. We also identified some fungal (yeast) sequences in a ZR rat. It is not clear whether they are parasitic or endosymbiotic species, but this suggests that 18S Illumina sequencing may enable us to investigate associations between helminths and protozoan parasites or parasites and other eukaryotic micro-organisms.

## Conclusions

Studying the diversity of parasites has been recognised as an interesting and useful approach in several research fields, such as those investigating the evolution of life, ecosystem fractions and invasion and migration of emerging diseases. However, the difficult and time-consuming processes required to identify parasites have restricted analysis covering a wide range of parasite species or dealing many samples. In this study, we showed the power and usefulness of 18S rDNA-based metagenomics in the investigation of parasite diversity. We also showed this approach still needs improvements in database completeness and read length in order to classify various parasites into a sufficient level. This approach with those improvements will enable us to analyse a large number of samples in a high throughput manner and can be the next standard to investigate parasite diversity.

## Supporting Information

Figure S1 **Maximum likelihood phylogenies based on 18S rDNA sequences to show relationships of the isolated worms with other nematode or cestode species/isolates.** Selected nematode or cestode species were used in each of five trees; [A] with the sequences from morph A (A1) and morph C (C3), [B] morph B (B1, B2) and morph C (C1, C2), [C] morph D (D1, D2) and morph E (E1), [D] morph F (F1), [E] morph G (G1). Sequences obtained in this study were boxed in red, in which sequence group IDs (shown in Table 4) and individual sequence IDs (in parentheses) were given. Bootstrap values greater than 60% are shown on appropriate nodes. Genbank accession numbers were shown after the species names in each tip label.
(PDF)

Table S1 **QIIME classification taxonomy summary (Level 6).**
(XLSX)
