# Supplementary material for: Assessment of Helminth Biodiversity in Wild Rats Using 18S rDNA Based Metagenomics
Source: PLoS One. 2014 Oct 23;9(10):e110769. doi: 10.1371/journal.pone.0110769 (PMC4207705; doi:10.1371/journal.pone.0110769)
Supplement: Figure S1 — Maximum likelihood phylogenies based on 18S rDNA sequences to show relationships of the isolated worms with other nematode or cestode species/isolates. Selected nematode or cestode species were used in each of five trees; [A] with the sequences from morph A (A1) and morph C (C3), [B] morph B (B1, B2) and morph C (C1, C2), [C] morph D (D1, D2) and morph E (E1), [D] morph F (F1), [E] morph G (G1). Sequences obtained in this study were boxed in red, in which sequence group IDs (shown in Table 4) and individual sequence IDs (in parentheses) were given. Bootstrap values greater than 60% are shown on appropriate nodes. Genbank accession numbers were shown after the species names in each tip label. (PDF) [file pone.0110769.s001.pdf]

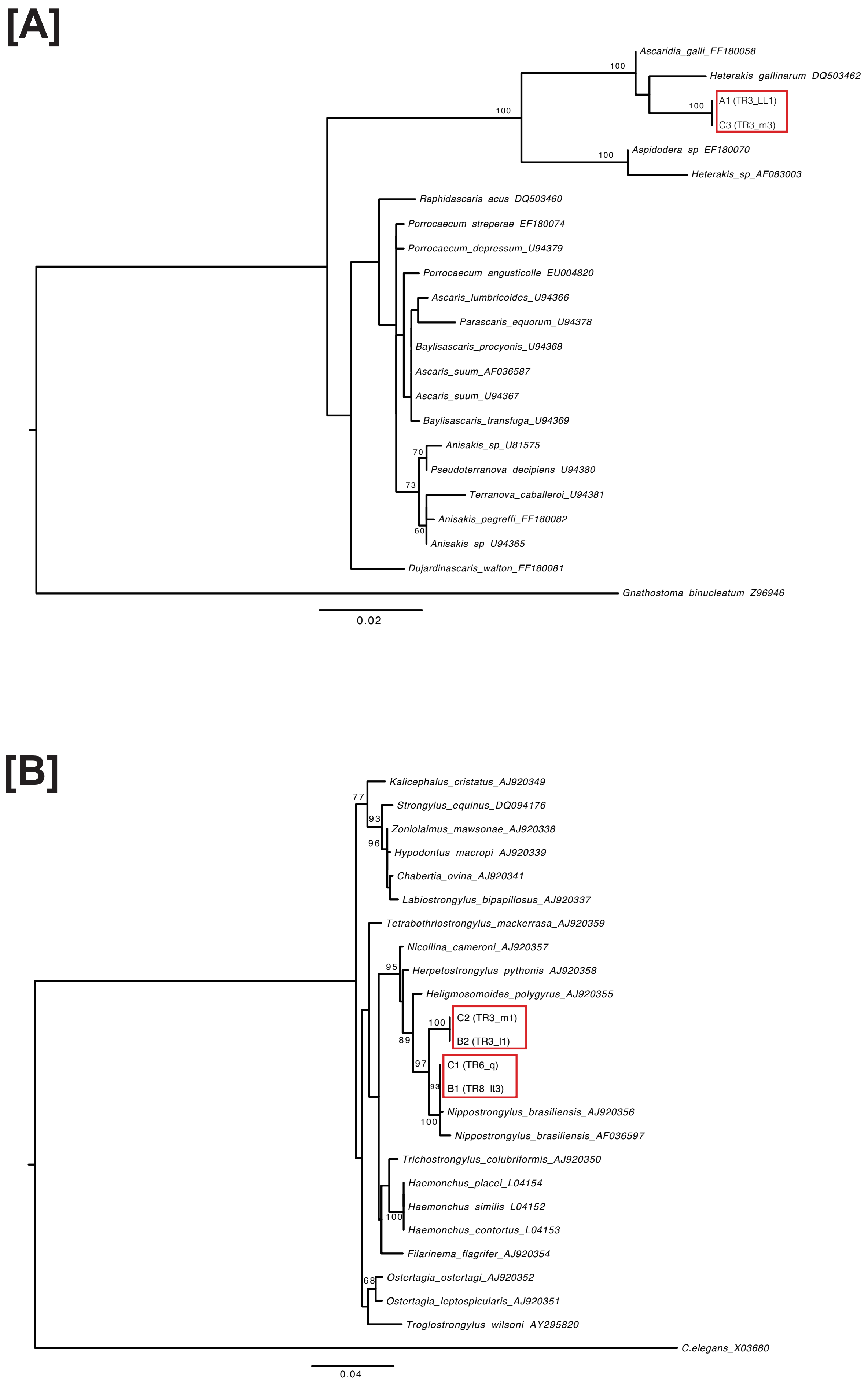
[A]
Ascaridia_galli_EF180058
Heterakis_gallinarum_DQ503462
A1 (TR3_LL1)
C3 (TR3_m3)
Aspidodera_sp_EF180070
Heterakis_sp_AF083003
Raphidascaris_acus_DQ503460
Porrocaecum_streperae_EF180074
Porrocaecum_depressum_U94379
Porrocaecum_angusticolle_EU004820
Ascaris_lumbricoides_U94366
Parascaris_equorum_U94378
Baylisascaris_procyonis_U94368
Ascaris_suum_AF036587
Ascaris_suum_U94367
Baylisascaris_transfuga_U94369
Anisakis_sp_U81575
Pseudoterranova_decipiens_U94380
Terranova_caballeroi_U94381
Anisakis_pegreffi_EF180082
Anisakis_sp_U94365
Dujardinascaris_walton_EF180081
Gnathostoma_binucleatum_Z96946
100
100
100
100
70
73
60
0.02
[B]
Kalicephalus_cristatus_AJ920349
Strongylus_equinus_DQ094176
Zoniolaimus_mawsonae_AJ920338
Hypodontus_macropi_AJ920339
Chabertia_ovina_AJ920341
Labiostrongylus_bipapillosus_AJ920337
Tetrabothriostrongylus_mackerrasa_AJ920359
Nicollina_cameroni_AJ920357
Herpetostrongylus_pythonis_AJ920358
Heligmosomoides_polygyrus_AJ920355
C2 (TR3_m1)
B2 (TR3_l1)
C1 (TR6_q)
B1 (TR8_lt3)
Nippostrongylus_brasiliensis_AJ920356
Nippostrongylus_brasiliensis_AF036597
Trichostrongylus_colubriformis_AJ920350
Haemonchus_placei_L04154
Haemonchus_similis_L04152
Haemonchus_contortus_L04153
Filarinema_flagrifer_AJ920354
Ostertagia_ostertagi_AJ920352
Ostertagia_leptospicularis_AJ920351
Troglostrongylus_wilsoni_AY295820
C.elegans_X03680
77
93
96
95
89
100
97
93
100
100
68
0.04

[C]

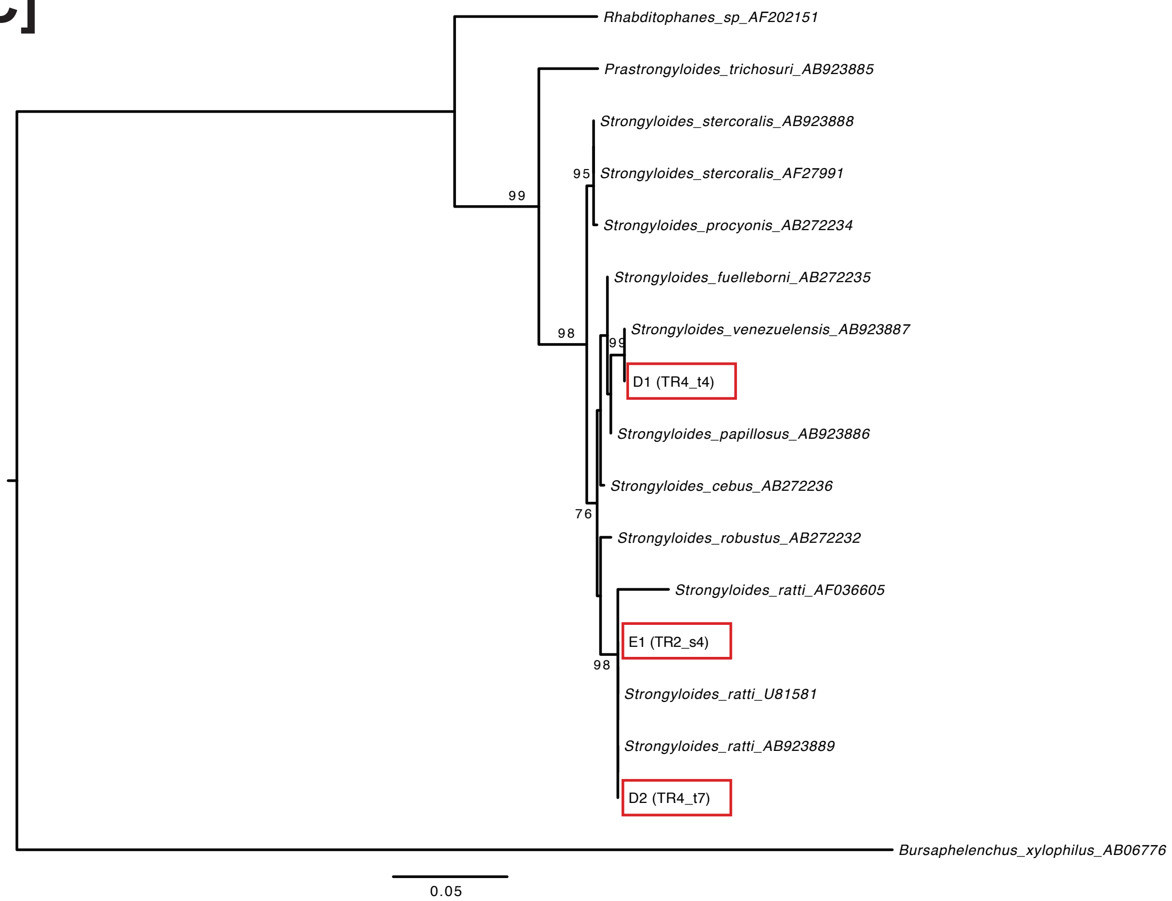
Rhabditophanes_sp_AF202151
Prastrongyloides_trichosuri_AB923885
Strongyloides_stercoralis_AB923888
95
Strongyloides_stercoralis_AF27991
99
Strongyloides_procyonis_AB272234
Strongyloides_fuelleborni_AB272235
Strongyloides_venezuelensis_AB923887
98
99
D1 (TR4_t4)
Strongyloides_papillosus_AB923886
Strongyloides_cebus_AB272236
76
Strongyloides_robustus_AB272232
Strongyloides_ratti_AF036605
E1 (TR2_s4)
98
Strongyloides_ratti_U81581
Strongyloides_ratti_AB923889
D2 (TR4_t7)
Bursaphelenchus_xylophilus_AB06776
0.05

[D]

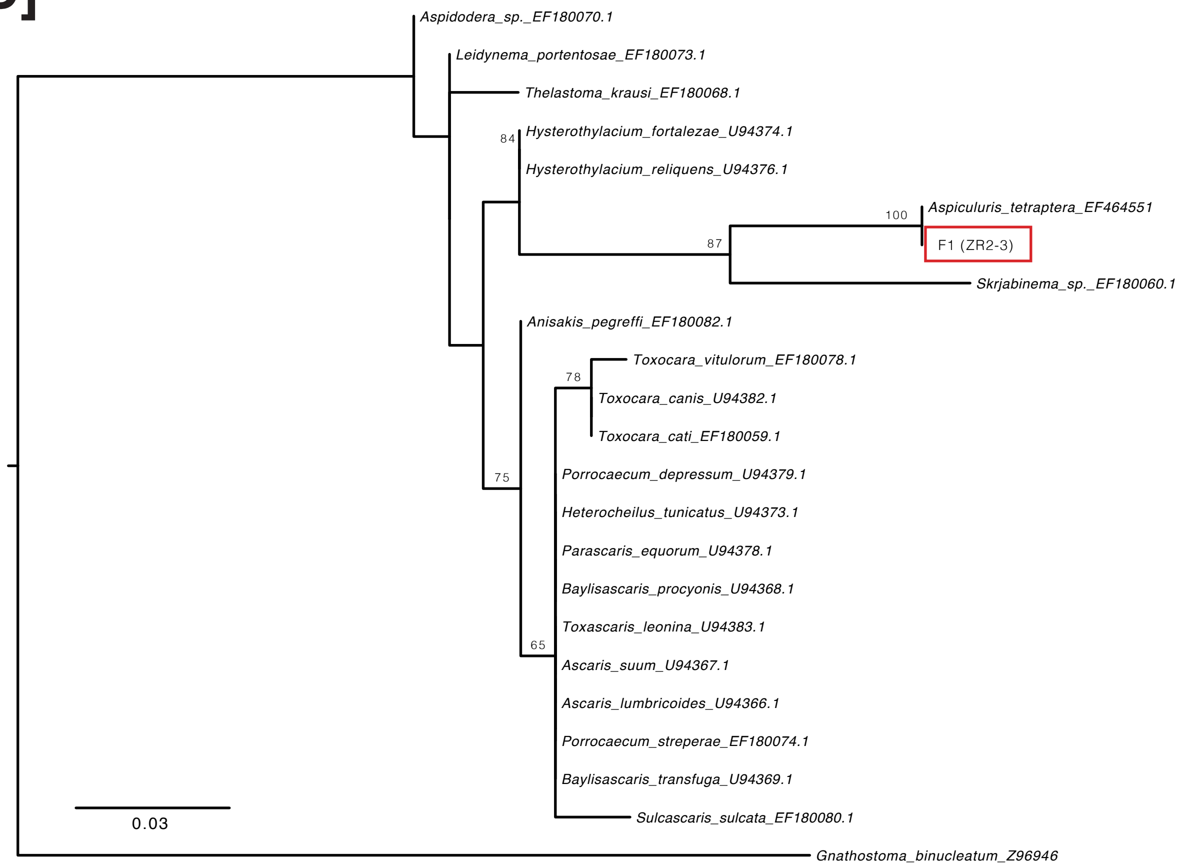
Aspidodera_sp._EF180070.1
Leidynema_portentosae_EF180073.1
Thelastoma_krausi_EF180068.1
84
Hysterothylacium_fortalezae_U94374.1
Hysterothylacium_reliquens_U94376.1
100
Aspiculuris_tetraptera_EF464551
87
F1 (ZR2-3)
Skrjabinema_sp._EF180060.1
Anisakis_pegreffi_EF180082.1
Toxocara_vitulorum_EF180078.1
78
Toxocara_canis_U94382.1
Toxocara_cati_EF180059.1
75
Porrocaecum_depressum_U94379.1
Heterocheilus_tunicatus_U94373.1
Parascaris_equorum_U94378.1
Baylisascaris_procyonis_U94368.1
Toxascaris_leonina_U94383.1
65
Ascaris_suum_U94367.1
Ascaris_lumbricoides_U94366.1
Porrocaecum_streperae_EF180074.1
Baylisascaris_transfuga_U94369.1
0.03
Sulcascaris_sulcata_EF180080.1
Gnathostoma_binucleatum_Z96946

[E]

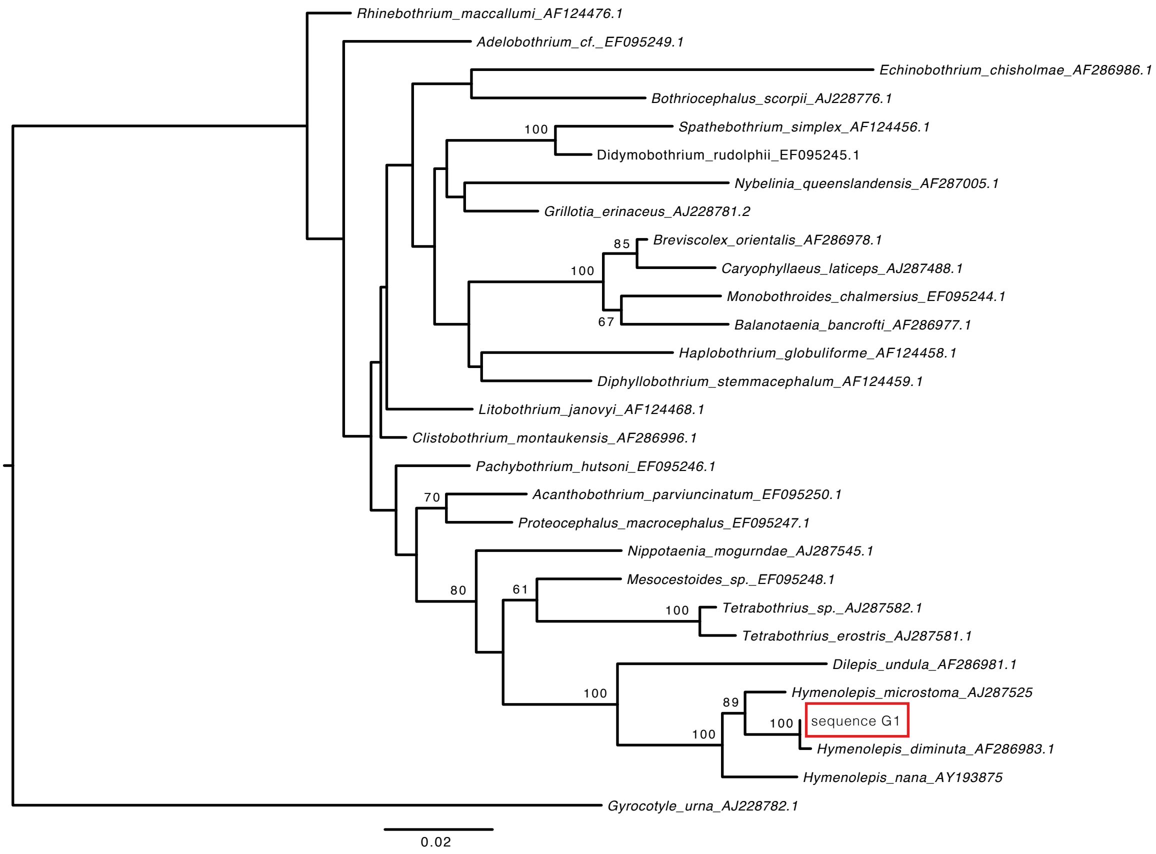

Figure S1. Maximum likelihood phylogenies based on 18S rDNA sequences to show relationships of the isolated worms with other nematode or cestode species/isolates. Selected nematode or cestode species were used in each of five trees; [A] with the sequences from morph A (A1) and morph C (C3), [B] morph B (B1, B2) and morph C (C1, C2), [C] morph D (D1, D2) and morph E (E1), [D] morph F (F1), [E] morph G (G1). Sequences obtained in this study were boxed in red, in which the sequence group IDs (shown in Table 3B) and individual sequence IDs (in parentheses) were given. Bootstrap values greater than 60% are shown on appropriate nodes. Genbank accession numbers were shown after the species names in each tip label.
